# Coronin 1a-mediated F-actin disassembly controls effector function in murine neutrophils

**DOI:** 10.1016/j.redox.2025.103618

**Published:** 2025-03-26

**Authors:** Anton Shaverskyi, Jan Hegermann, Korbinian Brand, Kyeong-Hee Lee, Niko Föger

**Affiliations:** a Inflammation Research Group, Institute of Clinical Chemistry, Hannover Medical School, Hannover, Germany; b Institute of Clinical Chemistry, Hannover Medical School, Hannover, Germany; c Central Research Facility Electron Microscopy, Hannover Medical School, Hannover, Germany

**Keywords:** Neutrophil effector function, Oxidative burst, NADPH oxidase, Actin cytoskeleton, Coronin1a

## Abstract

The double-edged role of neutrophils in effective host defense and harmful pathology is an emerging topic in clinical research. Neutrophils release highly potent antimicrobial granule compounds and reactive oxygen species (ROS) that can also be detrimental to the host and promote inflammatory diseases and cancer. Here we show that disassembly of F-actin greatly facilitates ROS production and degranulation in neutrophils. Utilizing neutrophils from Coronin 1a (Coro1a)-deficient mice, our data reveal that the actin-regulatory protein Coro1a controls this spatial F-actin deconstruction and concomitantly forms a signaling complex with Rac-GTPases, thereby promoting activation and translocation of Rac to the membrane during neutrophil activation. This functional activity of Coro1a was critical for neutrophil granule exocytosis and the activation of the NADPH oxidase complex. Consistent with these findings, impaired ROS production in *Coro1a*-deficient neutrophils was rescued by pharmacological promotion of actin depolymerization or activation of Rac. Together, our findings suggest that the Coro1a/Rac signaling hub acts as a central regulatory element that coordinates actin cytoskeletal reorganization required for the execution of neutrophil effector functions. Since Coro1a is highly conserved between mice and humans and associated with human immunodeficiency, our results are also relevant for human biomedical studies.

## 1 Introduction

Neutrophils are the most abundant circulating immune cells in the human bloodstream. Due to their high number and great capacity to rapidly traffic to sites of infection in response to innate sensing, neutrophils are recognized as an indispensable first line of immune defense [1]. However, increasing clinical evidence also points out harmful sides of neutrophils that can actively contribute to pathological conditions in inflammatory, autoimmune and malignant diseases [2,3]. A powerful set of neutrophil effector functions, such as the release of granular enzymes, production of reactive oxygen species (ROS) and formation of neutrophil extracellular traps (NETs) is highly effective in combating pathogens but can also injure host cells, resulting in collateral tissue damage, defective healing processes, and excessive inflammation, which is particularly critical in the context of chronic inflammation and autoimmunity. In addition, mediators released by neutrophils can also promote tumor growth and angiogenesis [4,5].

Neutrophils generate large amounts of highly microbicidal ROS via activation of the NADPH oxidase, a multi-subunit membrane-associated enzyme complex that is assembled at the plasma membrane or at phagosomal membranes following neutrophil stimulation [6]. The NADPH oxidase complex is composed of the Rho GTPase Rac2 and five phox subunits. Under resting conditions, the multidomain regulatory subunits p40^phox^, p47^phox^, and p67^phox^ exist as a complex in the cytosol. After stimulation, this cytosolic complex migrates to the membrane where it subsequently interacts with Cyt_b558_, a heterodimer of two membrane-bound subunits, p22^phox^ and gp91^phox^, that forms the catalytic core of the NADPH oxidase [7,8]. Assembly and activation of the NADPH oxidase complex is dependent on phosphorylation of regulatory phox subunits and activation of Rac2 to its GTP-bound state [7,9]. Activated NADPH oxidase then catalyzes the generation of superoxide anions that can be converted to other toxic reactive oxygen species.

Granule exocytosis, also known as degranulation, is another essential effector function of neutrophils. Upon activation, neutrophils release a plethora of preformed antimicrobial mediators and proteases from distinct granule subsets in the process of degranulation, either secreting intragranular effector molecules intracellularly by merging granules with the forming phagosome or extracellularly by fusing with the plasma membrane [10]. The release of granule-derived mediators is strictly controlled by receptor-coupled activation of intracellular signal transduction pathways. Exocytosis of granules occurs in several discrete steps, including intracellular trafficking of granules from the cytoplasm to the target membrane and subsequent vesicle binding and docking, followed by fusion of the granules with the target membrane, which occurs rapidly and develops a reversible fusion pore structure between the granule and the target membrane [[11], [12], [13]].

Activation-induced dynamic changes of the actin cytoskeleton play a critical role in the regulation of immune cell function and dysregulations of the actin cytoskeleton are associated with various immunological disorders and immunodeficiencies [[14], [15], [16]]. Actin cytoskeletal remodeling appears to be involved in various neutrophil effector functions, such as, the mobilization of all granule subtypes and NET formation in neutrophils [12,[17], [18], [19]]. Also, cytosolic subunits of the NADPH oxidase complex, p40^phox^, p47^phox^ and p67^phox^, have been found to be associated with the actin cytoskeleton in neutrophils [[20], [21], [22], [23]].

Coronins are evolutionary conserved actin-regulatory proteins that participate in F-actin remodeling [24,25]. Among the 7 mammalian coronin family members, Coronin 1a (Coro1a) is mainly restricted to cells of the hematopoietic lineage. Deletion or mutation of human CORO1A causes severe combined immunodeficiency (SCID), that has been largely attributed to peripheral T cell lymphopenia, leaving affected patients vulnerable to life-threatening viral and microbial infections [[26], [27], [28], [29]]. A similar reduction in peripheral T cells was also observed in Coro1a-mutant mice [[30], [31], [32], [33]], highlighting the high similarity between human and mouse Coro1a. Coro1a-participates in the turnover of F-actin [24] and its actin-regulatory activity has been implicated in the function of T cells and other immune cell types, such as NK cells and mast cells [30,31,34,35]. Notably, expression of Coro1a is especially high in neutrophils, where it accounts for ∼1.56 % of total protein [36], suggesting a fundamental requirement of this protein for neutrophil function.

Excessive ROS production and granule release by neutrophils is a common feature in various inflammatory diseases. Thus, a better understanding of the basic mechanisms that regulate the assembly and activity of the NADPH oxidase complex and neutrophil degranulation is central to the development of innovative treatment strategies for patients with neutrophil-mediated immunopathology. Employing pharmacological modulation of actin polymerization and *Coro1a*-deficient mice, the current study investigated the physiological impact of actin cytoskeletal dynamics on neutrophil effector function. In addition, we explored underlying molecular mechanisms of actin cytoskeletal remodeling that contribute to the activation of the NADPH oxidase complex and granule release in neutrophils.

## 2 Results

**Fig. 1:**
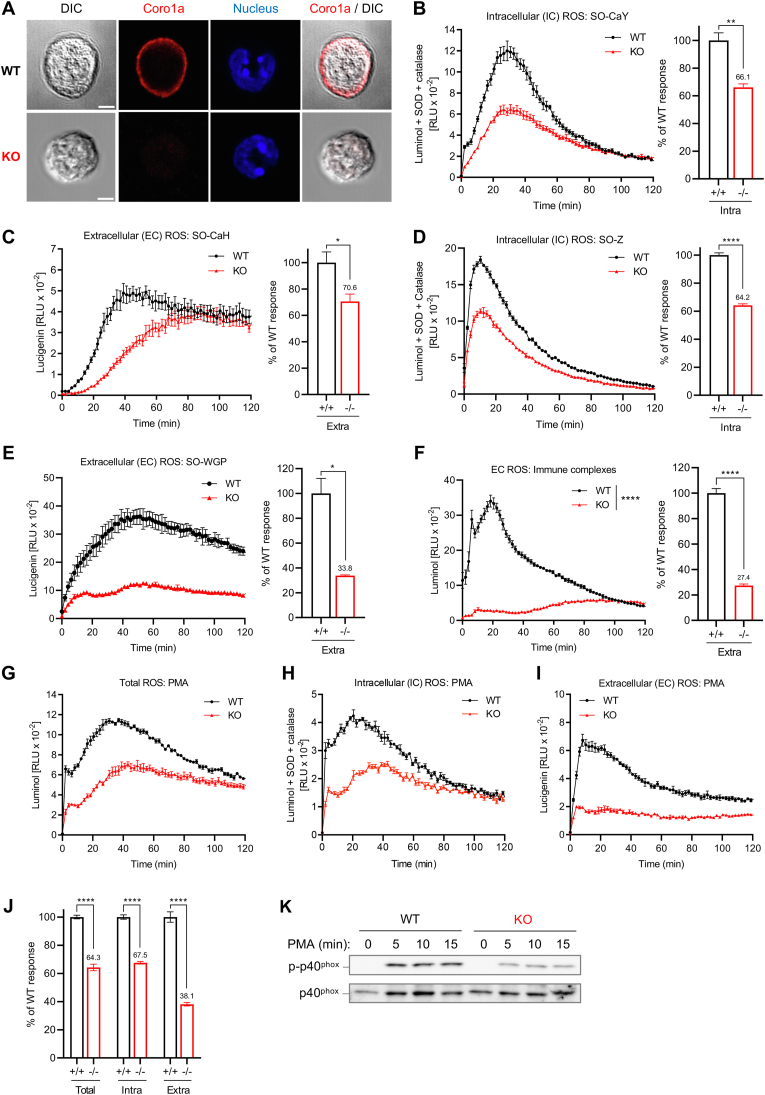
Impaired ROS production in neutrophils deficient for the actin-regulatory protein Coro1a **(A**) Confocal microscopy of WT and *Coro1a*^−/−^ (KO) neutrophils showing the subcellular localization of Coro1a. Neutrophils were fixed, permeabilized and stained for Coro1a together with DAPI (nucleus). DIC, differential interference contrast. Scale bar, 3 μm. **(B**–**J)** ROS generation in WT and *Coro1a*^−/−^ (KO) neutrophils. **(B, D)** Production of intracellular ROS in response to ingestible, serum opsonized **(B)***C. albicans* yeast (SO–CaY) or **(D)** zymosan particles (SO-Z) measured with luminol chemiluminescence in the presence of superoxide dismutase (SOD) and catalase. **(C, E, F)** Production of extracellular ROS in response to non-ingestible **(C)** serum-opsonized *C. albicans* hyphae (SO–CaH), **(E)** serum-opsonized whole glucan particles (SO-WGP; filtered to have Ø ≥ 20 μm), or **(F)** immobilized BSA/anti-BSA immune complexes using (**C, E**) lucigenin or (**F**) luminol chemiluminescence assays. Left: ROS response kinetics; Right: normalized integrated ROS signals. **(G**–**J)** Production of **(G)** total, **(H)** intracellular, and **(I)** extracellular ROS in response to stimulation with PMA (100 ng/ml) measured with chemiluminescence assays. **(J)** Normalized integrated PMA-induced ROS signals. **(K)** Western-blot showing PMA-induced phosphorylation of p40phox in WT and *Coro1a*^−/−^ (KO) neutrophils. **(B–I)** Data show mean ± SEM (n = 2–4). Unpaired Student's t-test: ∗ <0.05, ∗∗ <0.01, ∗∗∗∗ <0.0001. Data are representative for at least 4 independent experiments.

To explore the biological impact of the actin-regulatory protein Coronin 1a (Coro1a) on neutrophil effector function, we first analyzed the expression pattern of coronins. Real-time PCR analysis revealed that the Coro1a transcript had the highest expression level amongst coronin family members in murine neutrophils (Fig. S1A), which is consistent with the previously reported high expression of Coro1a in human neutrophils [36]. Coro1a expression in mouse neutrophils was further confirmed on the protein level by western blotting (Fig. S1B). Noteworthy, Coro1a-deficiency did not result in compensatory upregulation of any of the other immunologically relevant coronin family proteins (Fig. S1B). In accordance with previous observations in T cells, macrophages and mast cells [30,34,37], Coro1a was predominantly localized to the cell cortex beneath the plasma membrane in neutrophils (Fig. 1A).

### 2.1 Impaired NADPH oxidase-mediated ROS production in neutrophils deficient for the actin-regulatory protein Coro1a

Analysis of neutrophil development in the bone marrow indicated normal neutrophil differentiation in *Coro1a*^−/−^ mice (Figs. S1C–E). *Coro1a*^−/−^ mice showed normal frequencies of neutrophil progenitors (G1), pre-neutrophils (G2), immature neutrophils (G3), and mature neutrophils (G4) in the bone marrow, with only a slight accumulation of mature neutrophils (G4) that most likely can be attributed to reduced egress related to impaired trafficking [38].

A central antimicrobial effector function of neutrophils is the production of reactive oxygen species (ROS), also known as oxidative burst formation, that is mediated by the NADPH oxidase complex. Besides its potent anti-bacterial effects, ROS generation by neutrophils is also key to the elimination of pathogenic fungi. Indeed, fungal infections, including infections with *Candida* spp., pose a major threat to the survival of patients with chronic granulomatous disease (CGD), an immune disorder that is caused by defective NADPH oxidase activity [39]. We thus checked ROS generation in *Coro1a*^−/−^ neutrophils in response to a live fungus, *C. albicans* SC513, a well-studied clinical isolate that belongs to a closely related *C. albicans* clade, cumulatively responsible for ∼75 % of all *Candida* infections worldwide [40]. *C. albicans* is a dimorphic fungus, which grows as small yeast or as large invasive filamentous hyphae. Importantly, intracellular ROS to serum-opsonized small *C. albicans* yeast as well as extracellular ROS to serum-opsonized large *C. albicans* hyphae were both significantly reduced in *Coro1a*^−/−^ neutrophils compared to WT controls as assessed by time kinetic measurements of the oxidation of chemiluminescent probes (Fig. 1B and C).

To better differentiate between effects of different stimulus sizes and thus between phagocytosis-dependent and -independent mechanisms and also considering that fungal virulence has evolved strategies to detoxify ROS [41], we next checked ROS responses to isolated fungal β-glucan structures: zymosan, and whole glucan particles (WGP). These particles differ in size, with zymosan's diameter ranging from 3 to 4 μm and being phagocytosable, whereas WGP was filtered to be larger than 20 μm and thus non-ingestible. Intracellular ROS production to serum-opsonized small zymosan particles was reduced by more than one third in *Coro1a*^−/−^ neutrophils (Fig. 1D), while, strikingly, extracellular ROS in response to non-ingestible serum-opsonized large WGP was even impaired by >60 % in the absence of Coro1a (Fig. 1E). Extracellular ROS generation induced by immobilized immune complexes, another phagocytosis-independent model, was also substantially reduced by ca. 70 % in *Coro1a*^−/−^ neutrophils compared to WT controls (Fig. 1F), thus providing further evidence for phagocytosis-independent impaired ROS-generation upon deletion of Coro1a.

Furthermore, total ROS production, as well as intracellular and particularly extracellular ROS production were all significantly reduced in *Coro1a*^−/−^ neutrophils compared to wild types (WT) in response to the soluble stimulus PMA (Fig. 1G–J). Dose-response experiments showed preservation of defective ROS production upon *Coro1a*-deficiency over a wide range of PMA concentrations, with similar gaps between ROS amplitudes of WT and *Coro1a*^−/−^ neutrophils (Fig. S2A).

*Coro1a*^−/−^ neutrophils exhibited similarly impaired ROS production when using an alternative method to assess intracellular ROS by the flow cytometry-based dihydrorhodamine 123 (DHR123) assay (Figs. S2B and C). Defective ROS generation in *Coro1a*^−/−^ neutrophils was clearly associated with the NADPH oxidase pathway, as treatment with the NADPH oxidase inhibitor diphenyleneiodonium (DPI) confirmed that ROS produced by NADPH oxidase rather than ROS generated by mitochondria was measured (Fig. S2C). Flow cytometric live/dead discrimination also confirmed that impaired ROS production in *Coro1a*^−/−^ neutrophils was not due to changes in cell viability. As an additional control, all components of the NADPH oxidase complex were expressed at comparable levels in *Coro1a*^−/−^ and WT neutrophils (Fig. S2D).

Consistent with previous reports that have implicated Coro1a in phagocytic processes [36,42,43], *Coro1a*^−/−^ neutrophils showed reduced phagocytic uptake of FITC-labeled serum-opsonized zymosan particles (Fig. S3). While the role of Coro1a in phagocytosis could potentially contribute to ROS production in response to stimuli that can be phagocytosed, impaired ROS production by *Coro1a*^−/−^ neutrophils was particularly observed with various phagocytosis-independent stimuli. Our data, thus, strongly argue for a general defect in ROS generation in *Coro1a*^−/−^ neutrophils that is independent of their phagocytic capacity. Noteworthy, impaired oxidative burst formation was not observed in another *Coro1a*-deficient mouse strain that was generated via a different targeting strategy [44]. Together, based on a wide range of different stimulation conditions using various soluble and particulate stimuli, we provide ample experimental evidence for a critical involvement of the actin regulatory protein Coro1a in the regulation of NADPH oxidase-mediated ROS production in neutrophils.

Activation of the neutrophil NADPH oxidase is tightly controlled by phosphorylation of phox protein subunits [9]. While phosphorylation of p47^phox^ at Ser-328 was not substantially affected by Coro1a-deficiency (Fig. S2E), activation-induced phosphorylation of p40^phox^ at Thr-154, which is physiologically required for complete activation of the NADPH oxidase [45], was clearly reduced in *Coro1a*^−/−^ neutrophils (Fig. 1K). Thus, Coro1a is critically involved in the activation/formation of the NADPH oxidase complex in neutrophils.

### 2.2 Disassembly of F-actin promotes ROS generation in neutrophils

**Fig. 2:**
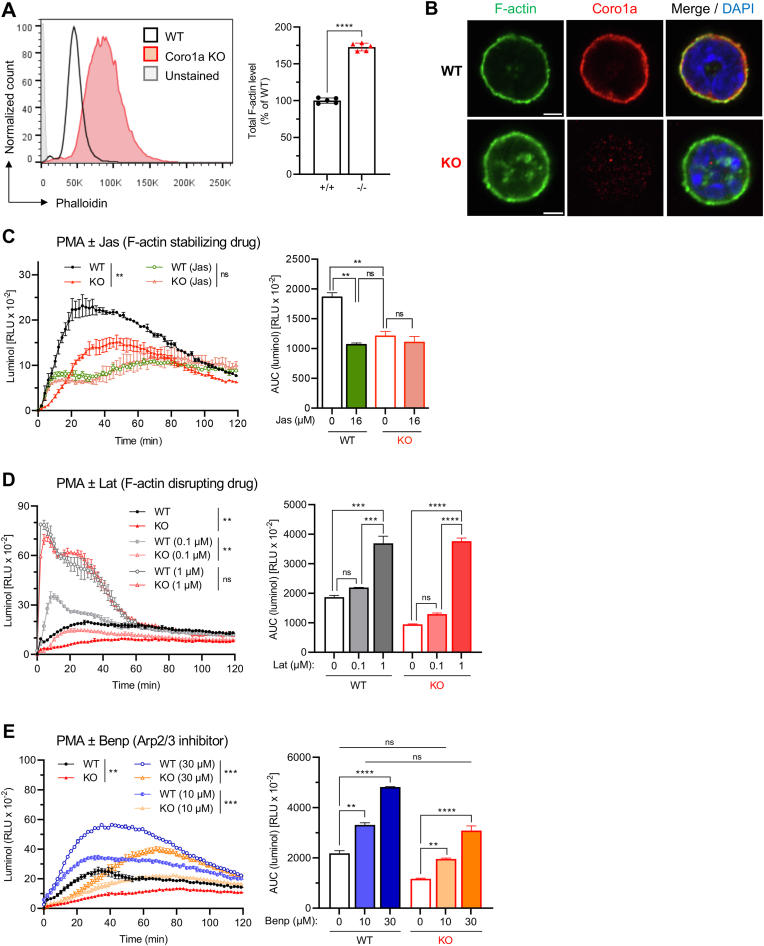
F-actin disassembly promotes ROS production in neutrophils **(A)** Cellular F-actin content detected by intracellular phalloidin staining and flow cytometric analysis. Left: representative FACS histogram; Right: relative F-actin level (n = 5). Unpaired Student's t-test. **(B)** Confocal microscopy of WT and *Coro1a*^−/−^ (KO) neutrophils showing staining for F-actin (phalloidin) and Coro1a. Nuclear staining: DAPI. Scale bar, 3 μm. **(C**–**E)** PMA (100 ng/ml)-induced ROS production in WT and *Coro1a*^−/−^ (KO) neutrophils pretreated for 60 min with the indicated concentrations of **(C)** jasplakinolide (Jas; actin-stabilizing agent), **(D)** latrunculin B (Lat; actin-depolymerizing agent); or **(E)** benproperine phosphate (Benp; Arp2/3-complex inhibitor). ROS production was measured with luminol chemiluminescence. Left: ROS response kinetics; Right: normalized integrated ROS signals. Data are mean ± SEM (n = 2). **(C**–**E)** 2-way ANOVA test (Tukey). ns > 0.05, ∗∗ <0.01, ∗∗∗ <0.001, ∗∗∗∗ <0.0001. Data are representative for at least 3 independent experiments.

Given the well-established role of coronin proteins in actin cytoskeletal regulation [24], we hypothesized that the regulatory function of Coro1a on neutrophil ROS generation may be related to effects on the actin cytoskeleton. Consistent with an actin-depolymerizing/destabilizing role of Coro1a, neutrophils deficient for Coro1a exhibited an increase in total cellular F-actin contents by >70 % compared to WT (Fig. 2A). Confocal microscopy showed that *Coro1a*^−/−^ neutrophils accumulate elevated levels of F-actin at the cell cortex and as cytoplasmic aggregates (Fig. 2B).

To gain understanding of whether actin cytoskeletal dysregulation in *Coro1a*^−/−^ neutrophils is responsible for defective ROS generation, we examined the effects of different actin modulatory drugs that either promote or inhibit actin polymerization on neutrophil oxidative burst formation. Mimicking enhanced F-actin levels of *Coro1a*^−/−^ neutrophils by treating WT neutrophils with jasplakinolide (Jas), an actin-polymerizing/stabilizing agent [46], resulted in a decrease in ROS production to a level similar to that of *Coro1a*-deficient neutrophils, while treatment with Jas did not further reduce ROS production of *Coro1a*-deficient cells (Fig. 2C). Conversely, treating neutrophils with the actin-depolymerizing reagent latrunculin B (Lat) resulted in a dose-dependent increase in cellular ROS generation (Fig. 2D). Noteworthy, upon pre-treatment with 1 μM Lat, PMA-induced ROS production peaked as a rapid signal that was comparable in both overall extend and kinetics between *Coro1a*^−/−^ and WT neutrophils (Fig. 2D). Thus, impaired ROS generation in *Coro1a*^−/−^ neutrophils can be overcome by promoting F-actin depolymerization, which counter-regulates the enhanced F-actin levels of these cells.

Coro1a interacts with the Arp2/3 complex in neutrophils [47] and has been described to inhibit Arp2/3 nucleating and branching activity [31,48]. Thus, to examine whether inhibiting Arp2/3 complex activity would reconstitute ROS production in *Coro1a*^−/−^ neutrophils, we treated neutrophils with benproperine phosphate (Benp), a small molecule inhibitor of Arp2/3 complex activity [49]. WT and *Coro1a*^−/−^ neutrophils exhibited increased ROS generation upon Bnp treatment and subsequent cell stimulation, with the overall amount of ROS generation being proportional to the Bnp concentration (Fig. 2E). Notably, pre-treating *Coro1a*^−/−^ neutrophils with moderate dose of Bnp (10 μM) lead to a cumulative ROS signal similar to WT neutrophils stimulated with PMA alone (Fig. 2E). Delayed ROS kinetics of Bnp-treated neutrophils may be attributed to the reported slowing down of the actin polymerization rate by Bnp [49]. As an important note, toxicity tests showed that non of the actin-modulatory drugs used in the above described experiments (Jas, Lat, and Bnp) had any notable effects on neutrophil viability (Fig. S5A).

Taken together, stabilization of the F-actin network, as also observed in *Coro1a*^−/−^ neutrophils, restricted oxidative burst, whereas, conversely, F-actin depolymerization promoted oxidative burst formation in neutrophils. Furthermore, mimicking Coro1a-mediated inhibition of Arp2/3 complex activity pharmacologically resulted in increased ROS production and rescued impaired oxidative burst in *Coro1a*^−/−^ neutrophils. Our data, thus, indicate that Coro1a-mediated actin cytoskeletal regulation, particularly its inhibitory function on Arp2/3 complex-mediated actin polymerization, controls neutrophil oxidative burst formation.

### 2.3 Coro1a-mediated spatial depolymerization of F-actin controls neutrophil granule release

**Fig. 3:**
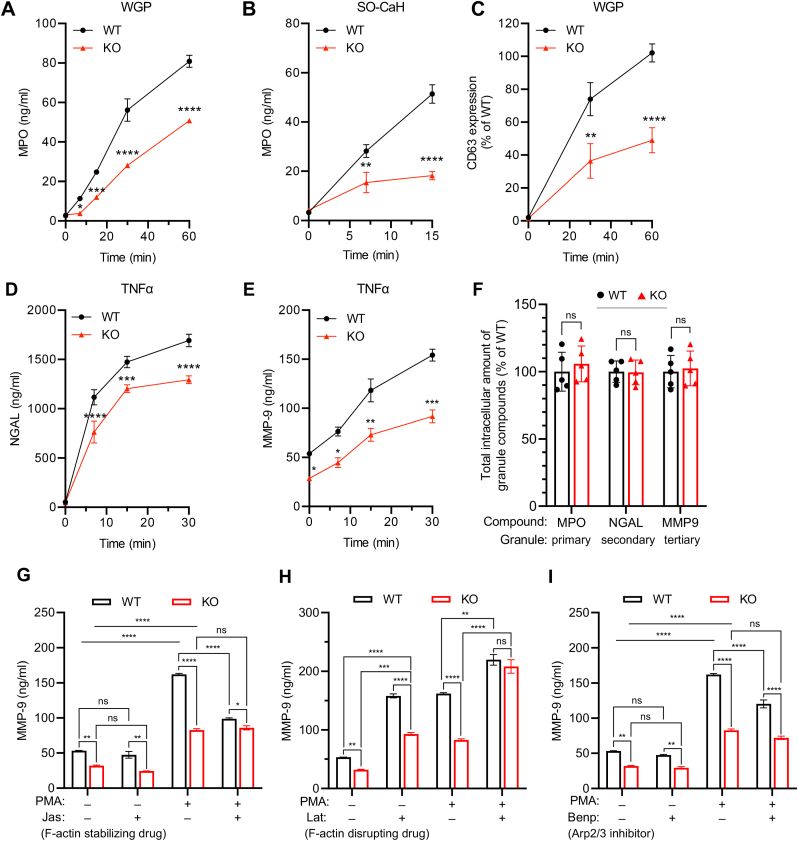
Enhanced F-actin levels in *Coro1a*-deficient neutrophils are associated with impaired granule release **(A-E)** Granule exocytosis (degranulation) by WT and *Coro1a*^−/−^ (KO) neutrophils. **(A, B)** Release of myeloperoxidase (MPO) from primary granules into culture supernatants upon stimulation of neutrophils with **(A)** serum-opsonized whole glucan particles (WGP) or **(B)** serum-opsonized *C. albicans* hyphae (SO–CaH). **(C)** Upregulation of CD63 (Lamp3) cell surface expression upon stimulation with WGP. **(D, E)** Release of **(D)** neutrophil gelatinase-associated lipocalin (NGAL) from secondary granules or **(E)** matrix metalloproteinase-9 (MMP-9) from tertiary granules into culture supernatants upon stimulation of neutrophils with TNFα. **(F)** Total amount of MPO, NGAL, and MMP-9 in WT and *Coro1a*^−/−^ (KO) neutrophils expressed in % of WT (n = 5). Unpaired Student's t-test. **(G**–**I)** PMA (100 ng/ml)-induced release of MMP-9 by WT and *Coro1a*^−/−^ (KO) neutrophils pretreated for 60 min with **(G)** 16 μM jasplakinolide (Jas; actin-stabilizing agent), **(H)** 1 μM latrunculin B (Lat; actin-depolymerizing agent); or **(I)** 30 μM benproperine phosphate (Benp; Arp2/3-complex inhibitor). Data are mean ± SEM (**A, D-E**: n = 3; **B, G-H**: n = 2; **C**: n = 7). **(A-E, G-H)** 2-way ANOVA. ns > 0.05, ∗ <0.05, ∗∗ <0.01, ∗∗∗ <0.001, ∗∗∗∗ <0.0001. Data are representative for at least 3 independent experiments.

Neutrophil degranulation, the release of a potent mix of pre-stored intragranular mediators that promote migration, enhance pathogen vulnerability, and cause microbicidal activity, is another key effector function of neutrophils. Toxic primary granules are relatively resistant to extracellular secretion and require large, non-phagocytosable prey for their release. Compared to WT controls, *Coro1a*^−/−^ neutrophils showed significantly reduced release of MPO from primary granules into culture supernatants upon stimulation with either WGP or *C. albicans* hyphae (Fig. 3A and B). Consistent with impaired exocytosis of primary granules, WGP-treated *Coro1a*^−/−^ neutrophils also displayed reduced upregulation of CD63 (Lamp3) cell surface expression, a transmembrane marker found exclusively in the membrane of primary granules (Fig. 3C). Exocytosis of NGAL from secondary granules and release of MMP-9 from tertiary granules was similarly decreased in *Coro1a*^−/−^ neutrophils in response to TNFα or PMA (Fig. 3D, E and Figs. S4A and B). Basal release of MMP-9 from tertiary granules under steady-state conditions was also reduced in *Coro1a*^−/−^ neutrophils (Fig. 3E). Importantly, total cellular amounts of MPO, NGAL, and MMP-9 were all comparable between unstimulated WT and *Coro1a*^−/−^ neutrophils (Fig. 3F), thus impaired mediator release by *Coro1a*^−/−^ neutrophils is not attributable to potential effects on granule cargo expression, but due to defects in the degranulation mechanism.

Similar to the before described effects on neutrophil ROS production, pharmacologic modulation of actin dynamics revealed that neutrophil degranulation is tightly associated with the remodeling of F-actin during cellular activation. Increasing cellular F-actin levels of WT neutrophils with jasplakinolide (Jas) lead to reduced release of MMP-9 into culture supernatants following PMA stimulation down to the level detected with *Coro1a*^−/−^ neutrophils treated with PMA alone (Fig. 3G). Of note, treatment with Jas did not further exacerbate already impaired granule exocytosis in *Coro1a*^−/−^ neutrophils, suggesting that maximal inhibition of degranulation by increased F-actin contents may already be reached in *Coro1a*^−/−^ neutrophils. On the other hand, promoting actin depolymerization by treatment with latrunculin B (Lat) triggered massive granule exocytosis, with WT and *Coro1a*^−/−^ neutrophils releasing similar amounts of MMP-9 following PMA stimulation (Fig. 3H). Incubation with Lat alone also resulted in enhanced release of MMP-9 at basal levels, although under these conditions the difference between WT and *Coro1a*^−/−^ neutrophils was still preserved. Our data, thus, show a strong dependence of neutrophil granule exocytosis on actin cytoskeletal regulation, with neutrophil degranulation generally being negatively controlled by cellular F-actin levels.

Pharmacological inhibition of Arp2/3 complex-mediated F-actin nucleation by treatment with benproperine phosphate (Benp), however, resulted in reduced secretion of MMP-9 by WT neutrophils, whereas effects on *Coro1a*^−/−^ neutrophils were negligible (Fig. 3I). These data indicate that neutrophil degranulation is both positively and negatively affected by actin polymerization dynamics, likely reflecting the high complexity of secretory granule release that involves multiple diverse processes.

**Fig. 4:**
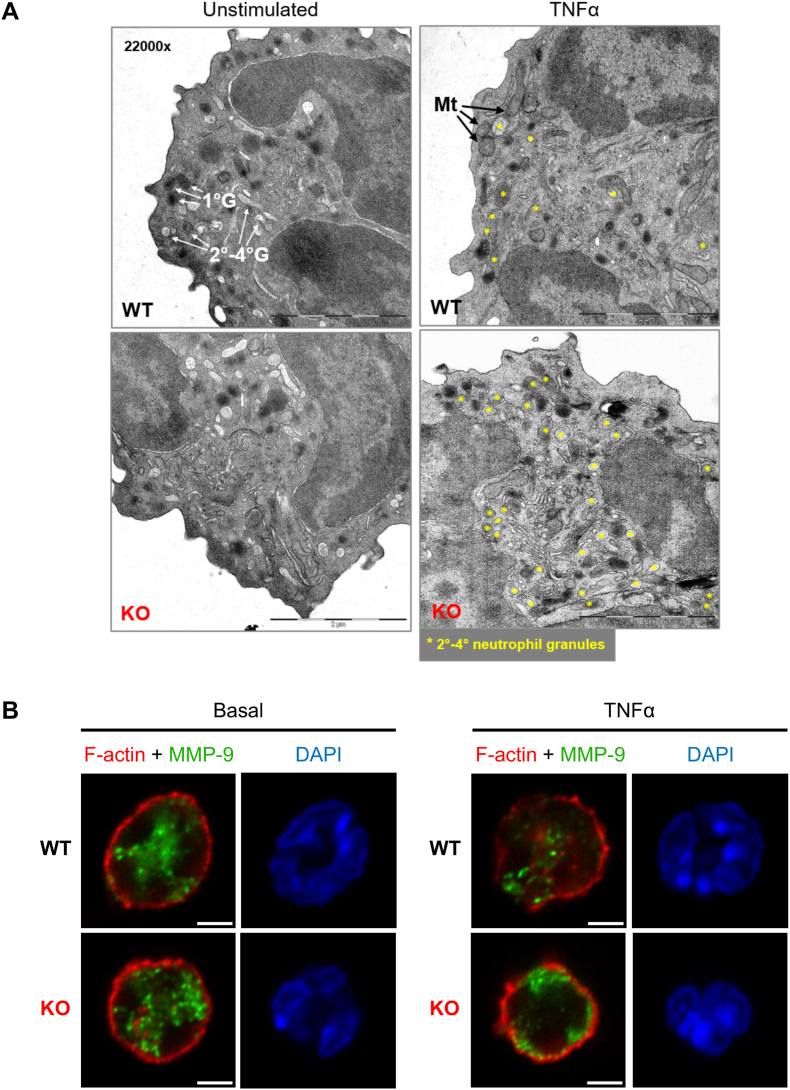
Local F-actin destruction is required for neutrophil granule exocytosis (**A**) Transmission electron microscopy (TEM) images of WT and *Coro1a*^−/−^ (KO) neutrophils either unstimulated or challenged with TNFα (20 ng/ml; 30 min). Micrograph labels: primary (1°G), secondary – quaternary (2°G – 4°G) granules, mitochondria (Mt). TNFα treated condition: non-primary granules are labeled with yellow asterisks. Scale bar, 2 μm; each micrograph is 4.5 μm × 4.5 μm, x22000 magnification. Images shown are representative of at least n = 50 neutrophils being analyzed from each condition. (**B**) Airyscan-enhanced confocal micrographs of WT and *Coro1a*^−/−^ (KO) neutrophils in basal state (left panel) or stimulated with 20 ng/ml of TNFα for 30 min (right panel). Neutrophils were fixed permeabilized and stained for F-actin (phalloidin) and MMP-9 together with DAPI (nucleus). Scale bar: 3 μm. Images shown are representative of neutrophils observed in 3 independent microscopy experiments. Each experiment consisted of analysis of 8–12 fields for every condition, with each field containing *ca.* 10–30 cells.

To obtain further insights into the cellular processes during neutrophil degranulation we turned to microscopic analyses. Transmission electron microscopy (TEM) showed that mature WT and *Coro1a*^−/−^ neutrophils have similar numbers and morphology of granules at steady-state (Fig. 4A, left and Fig. S4C). However, consistent with reduced degranulation upon Coro1a deletion, noticeable more secondary to quaternary granules were retained in the cytoplasm of TNFα-stimulated *Coro1a*^−/−^ neutrophils compared to WT neutrophils that had efficiently released these types of neutrophil granules (Fig. 4A, right and Fig. S4C). Use of immunofluorescence microscopy to directly visualize alterations/changes in the actin cytoskeleton and granule localization in resting and activated neutrophils provided further insights into the functional relation between impaired reorganization of F-actin and dysregulated granule release in *Coro1a*-deficient neutrophils. Following TNFα stimulation, WT neutrophils effectively cleared off cortical F-actin at the plasma membrane in exocytosis-active cellular loci allowing for release of the granule component MMP-9 into the extracellular space (Fig. 4B and Fig. S4D, WT). In striking contrast to WT cells, similarly treated *Coro1a*^−/−^ neutrophils were less efficient in dismantling the cortical F-actin barrier, hence most MMP-9-positive granules remaining trapped intracellularly, often accumulated close to cortical actin beneath the plasma membrane (Fig. 4B and Fig. S4D, KO).

Thus, neutrophil degranulation is tightly associated with the restructuring of the cortical F-actin network at the plasma membrane upon cellular stimulation. Our data further suggest that activation-induced local remodeling of cortical actin then allows for efficient docking and fusion of secretory granules with the plasma membrane.

### 2.4 Coro1a-associated protein complexes in neutrophils

**Fig. 5:**
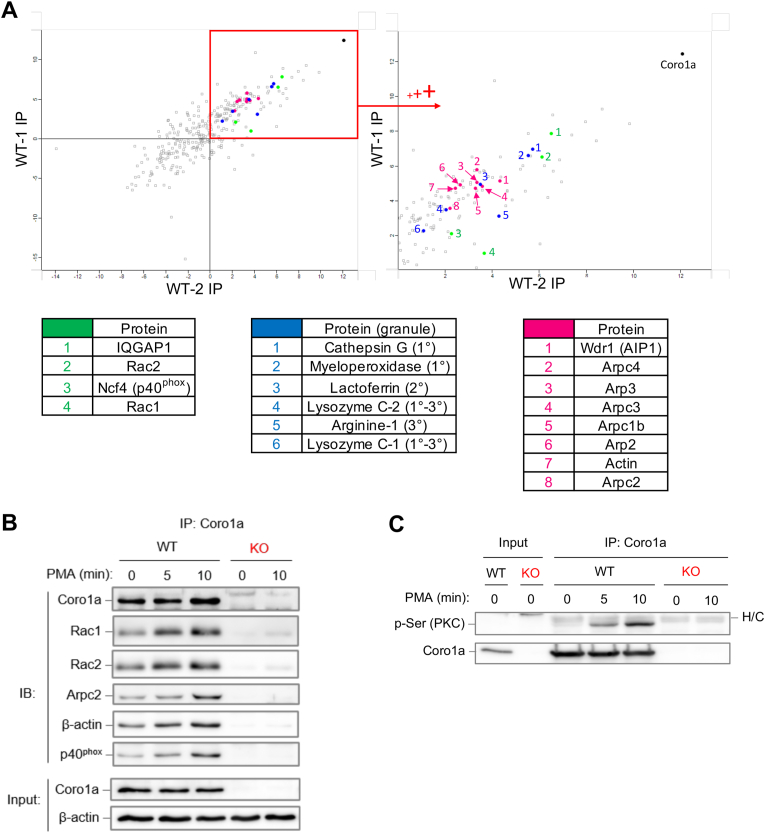
Identification of Coro1a-associated protein complexes in neutrophils **(A)** (Top left) Total scatter plot from co-immunoprecipitation with mass-spectrometry (Co-IP/MS) assay displaying proteins found co-precipitating with Coro1a in 2 biological replicates (WT-1 and WT-2 IP) of neutrophil lysates (plot was generated in Perseus software). (Top right) Magnification of the upper right quadrant of the scatter plot showing the most abundant proteins in the Coro1a-precipitates. (Bottom panels) Selected proteins from the Coro1a-precipitates are divided and color-coded into three groups (green: Ras proteins/NADPH oxidase; blue: neutrophil intragranular compounds (and granule subtype: primary – tertiary (1°–3°) granules); magenta: cytoskeletal proteins. IQGAP1, IQ motif-containing GTPase activating protein 1; Ncf4, neutrophil cytosolic factor 4 (*alias* p40^phox^); Wdr1, WD-repeat protein (*alias* AIP1, actin-interacting protein 1); Arp, actin-related protein; Arpc, Arp 2/3 complex proteins. **(B, C)** WT and *Coro1a*^−/−^ (KO) neutrophils were stimulated with PMA (100 ng/ml) for the indicated times. Cellular lysates were subjected to Coro1a-immunoprecipitation (IP) followed by Western blot analysis with the indicated Abs. (*p*-Ser (PKC): phospho-Serine (PKC substrate)). H/C: Ig heavy chain of immunoprecipitating Ab.

To identify coronin-associated protein complexes in neutrophils we performed Coro1a co-immunoprecipitation coupled to shotgun mass spectrometry-based proteomics. As shown in Fig. 5A, Coro1a co-precipitates were enriched in three groups of proteins: (1) Ras-related proteins & NADPH oxidase complex (green dots), (2) intragranular proteins (blue dots), and (3) proteins involved in actin cytoskeletal regulation (magenta dots). The first group of potential Coro1a interactors includes Rac1, Rac2, and the p40^phox^ subunit of the NADPH oxidase complex, as well as the IQ-motif containing GTPase-activating protein 1 (IQGAP1), a scaffold protein involved in the regulation of actin dynamics [50]. The second group contains components of primary to tertiary neutrophil granules including cathepsin G, MPO, and Lactoferrin, further supporting a regulatory role of Coro1a in the mobilization of these secretory granules. The third group of Coro1a-associated proteins are cytoskeletal proteins, including actin itself, Wdr1 (Aip1), a constituent of the ADF/cofilin pathway of actin disassembly [51] and components of the Arp2/3 complex, such as Arp2, Arp3 and Arpc2, the latter having been described as target for the inhibitory function of coronin in yeast [52]. These data further support a role of Coro1a in actin cytoskeletal regulation, particularly its involvement in F-actin depolymerization and regulation of Arp2/3 complex activity. Coro1a interaction with Rac1, Rac2, p40^phox^, and the Arp2/3 complex subunit Arpc2 was subsequently confirmed by co-immunoprecipitation and western blotting (Fig. 5B). Noteworthy, upon neutrophil stimulation Coro1a was phosphorylated on Ser residues (Fig. 5C) which correlated with increased association of Rac proteins, Arpc2, β-actin and p40phox upon neutrophil activation. The inducible association of these proteins with Coro1a suggests a functional involvement of theses protein interactions in neutrophil effector function.

### 2.5 Coro1a promotes activation and membrane translocation of Rac-GTPases

**Fig. 6:**
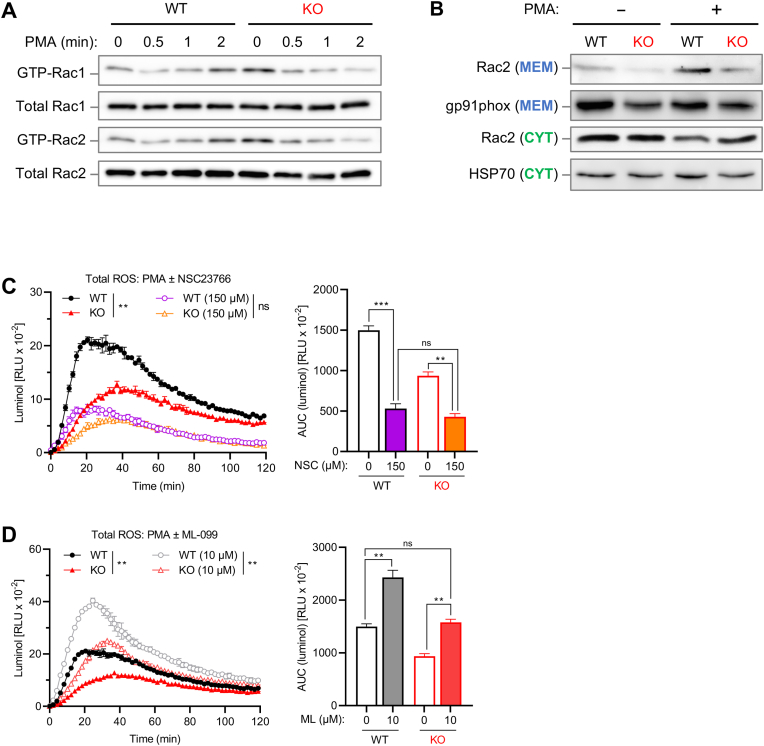
Coro1a-mediated activation and membrane translocation of Rac2 controls neutrophil ROS production **(A)** WT and *Coro1a*^−/−^ (KO) neutrophils stimulated with PMA (100 ng/ml) for the indicated times were analyzed for Rac1 and Rac2 activation by pull-down assay. Cell extracts were also analyzed for total amounts of Rac1 and Rac2. **(B)** PMA (100 ng/ml; 10 min)-induced translocation of Rac2 from the cytosol to the membrane was analyzed by subcellular fractionation assay. Gp91phox and HSP70 were used as loading controls for membrane and cytosolic fractions, respectively. **(C, D)** PMA (100 ng/ml)-induced ROS production in WT and *Coro1a*^−/−^ (KO) neutrophils pretreated for 60 min with the indicated concentrations of **(C)** NSC23766 (Rac inhibitor), or **(D)** ML-099 (pan Ras activator). ROS production was measured with luminol chemiluminescence. Left: ROS response kinetics; Right: normalized integrated ROS signals. **(C, D)** Data are mean ± SEM (n = 2). 2-way ANOVA test (Tukey). ns > 0.05, ∗∗ <0.01, ∗∗∗ <0.001, ∗∗∗∗ <0.0001. Data are representative for at least 3 independent experiments.

Rac2 activity is critical for NADPH oxidase-mediated oxidative burst formation in neutrophils [53]. Thus, given the observed role of Coro1a in neutrophil ROS production and its association with Rac-GTPases, we next examined the effects of Coro1a deficiency on Rac-activation. Noteworthy, activation of Rac1 and Rac2 proteins was rapidly induced in WT neutrophils within 2 min following cell stimulation, while *Coro1a*^−/−^ neutrophils instead displayed enhanced constitutive Rac activity at steady-state that was downregulated upon stimulation (Fig. 6A). Enhanced basal levels of active Rac (GTP-bound Rac) were similarly observed in coronin-deficient *D. discoideum* [54]. Subsequent subcellular fractionation assays showed that PMA-activation induced the translocation of Rac2 from the cytosolic fraction to the membranous fraction in WT neutrophils, whereas only minor membrane translocation of Rac2 was observed for *Coro1a*^−/−^ neutrophils (Fig. 6B). Thus, in addition to dysregulated Rac activation, *Coro1a*^−/−^ neutrophils also failed to efficiently translocate Rac from the cytosol to the membrane in response to cell stimulation.

Our study further investigated the functional relation between Coro1a and Rac in neutrophil oxidative burst by employing pharmacological modulation of Rac activity. Treatment with the Rac inhibitor NSC23766 resulted in reduced ROS production in both WT and *Coro1a*^−/−^ neutrophils down to a level that was comparable between the two genotypes (Fig. 6C), thus WT neutrophils being more sensitive to Rac inhibition compared to Coro1a-deficient neutrophils. Remarkably, the characteristic difference in kinetics between the different genotypes, with ROS production peaking ∼30 min earlier in WT neutrophils, was preserved. On the other hand, the converse approach, activation of Rac using the pan Ras-related activator ML-099, that potently activates Rac1 [55] efficiently boosted ROS production in both WT and *Coro1a*^−/−^ neutrophils (Fig. 6D). ML-099 treatment of *Coro1a*^−/−^ neutrophils rescued defective ROS production in these cells up to a level similar to control treated WT cells, while again differences in ROS kinetics were preserved. The observation that ML-099 increased ROS production in activated WT neutrophils to levels higher than in *Coro1a*^−/−^ neutrophils can likely be explained by Rac activation per se being not sufficient for effective ROS production, which may require additional Coro1a-mediated functions, such as membrane targeting of Rac, interaction of Coro1a with regulatory phox proteins and Coro1a-mediated F-actin remodeling during neutrophil activation. As control, Rac-modulating drugs did not affect cellular viability of either WT or *Coro1a*^−/−^ neutrophils (Fig. S5B).

In summary, our results from pharmacologic modulation of Rac activity together with functional data showing impaired activation and membrane translocation of Rac in *Coro1a*^−/−^ neutrophils strongly suggest that one critical mechanism by which Coro1a controls neutrophil oxidative burst is the regulation of the Rac pathway.

## 3 Discussion

The manifold activities of neutrophils in health and disease rely heavily on the release of large amounts of highly deleterious cytotoxic mediators during oxidative burst formation and granule exocytosis. Our study indicates that Cora1a-mediated spatial and temporal remodeling of the actin cytoskeleton is a decisive control mechanism for NADPH oxidase-induced ROS production and granule release in activated neutrophils.

Coro1a, one of the most abundant proteins in neutrophils, is involved in F-actin depolymerization by inhibiting Arp2/3-based actin assembly and branching [24,31,48,56]. We here show that Coro1a deficiency in neutrophils is associated with enhanced F-actin content, as it has been similarly described in murine T cells [30,31], and NK cells from patients with Coro1a deficiency [35], reflecting the indispensable role of Coro1a for F-actin depolymerization in these immune cell types. Importantly, dysregulation of the actin cytoskeleton in *Coro1a*-deficient neutrophils was critically related to impaired ROS production and degranulation.

Our study shows that pharmacological modulation of actin cytoskeletal dynamics results in remarkable changes in ROS production in activated neutrophils, with drug-induced F-actin depolymerization strongly potentiating oxidative burst generation, while, conversely, F-actin stabilization resulted in impaired ROS production and, thus, mimicked the phenotype of *Coro1a*^−/−^ cells. Defective ROS production in *Coro1a*^−/−^ neutrophils, in turn, could be pharmacologically compensated by F-actin-depolymerization agents. Our data, thus, clearly indicate that Coro1a-mediated dynamic restructuring of actin filaments is an essential prerequisite for efficient oxidative burst formation in neutrophils. Our observation that selective inhibition of the Arp2/3 complex rescues ROS production in *Coro1a*-deficient neutrophils further suggests that Coro1a regulates ROS generation via inhibition of Arp2/3-mediated *de novo* actin nucleation and branching.

Mechanistically, Coro1a is likely involved in the assembly and activation of the NADPH oxidase complex. First, phosphorylation of p40^phox^, a requirement for full NADPH oxidase activity [45], was substantially impaired in *Coro1a*^−/−^ neutrophils. Moreover, our data indicate that the function of Coro1a on NADPH oxidase-induced ROS production is also directly linked to the activation and subcellular relocalization of small Rac-GTPases. Coro1a was not only associated with Rac1 and Rac2 in neutrophils in an activation-dependent manner, but, importantly, Coro1a functionally controlled Rac activation and promoted the translocation of Rac2 from the cytosol to the membrane, which are both essential requirements for the formation of the NADPH oxidase complex [7,57]. These functional observations from primary neutrophils extend earlier reports that have implicated Coro1a in a regulatory feedback loop that controls membrane translocation and activation of Rac in an actin cytoskeleton-dependent manner during lammellipodium formation in immortalized cell lines [58,59]. Functional involvement of Rac-GTPases in Coro1a-dependent ROS regulation was further supported by our findings that wild type neutrophils exhibit relatively higher sensitivity to Rac inhibition compared to *Coro1a*-deficient cells, while promoting Rac activity pharmacologically largely restored ROS production in *Coro1a*-deficient neutrophils.

In addition to its interaction with Rac proteins that likely is mediated by the putative Cdc42-and Rac-interactive binding (CRIB) domain of Coro1a [60], Coro1a was also constitutively associated with other subunits of the NADPH oxidase complex, such as p40^phox^, and components of the Arp2/3 complex. Moreover, these associations were further enhanced during neutrophil activation and correlated with the phosphorylation status of Coro1a. Interactions of Coro1a with regulatory subunits of the NADPH oxidase-complex may account for additional (Rac-independent) ROS defects in *Coro1a*^−/−^ neutrophils, as modulation of Rac activity only rescued the overall extend of ROS formation, but not the delayed ROS kinetics of *Coro1a*-deficient neutrophils.

Together, our results indicate that Coro1a serves as a central regulatory hub that controls NADPH oxidase-complex formation and in turn neutrophil oxidative burst via the simultaneous coordination of F-actin remodeling and the activation and membrane targeting of Rac proteins. Based on our results and previous reports, the following tentative model may be proposed. Upon neutrophil stimulation, Coro1a, which is primarily localized to the cell cortex, gets activated and mediates local depolymerization of cortical F-actin at juxtamembrane areas by inhibiting Arp2/3-based actin nucleation/branching [24]. Coro1a simultaneously promotes the activation and membrane targeting of Rac-GTPases to the sites of Coro1a-mediated F-actin restructuring [58] that likely also contain Cyt_b588_ (p22^phox^ and gp91^phox^). Facilitated by the scaffolding ability of its β-propeller domain [61], Coro1a in conjunction with activated Rac may then coordinate further recruitment of regulatory phox subunits (p40^phox^, p47^phox^, and p67^phox^), leading to the formation of the fully active NADPH oxidase complex.

Coro1a-dependent F-actin remodeling was also critically involved in neutrophil granule exocytosis. Coro1a was found in association with several granule proteins, and components of the Arp2/3 complex and Coro1a deficiency resulted in defective release of primary, secondary, and tertiary granule subtypes. Microscopic analysis indicated that upon neutrophil activation, local depolymerization of cortical F-actin allows for granule release in wild type neutrophils, whereas in *Coro1a*-deficient neutrophils accumulation of a dense network of F-actin beneath the plasma membrane led to retention of most granules within the cytoplasm. Our data thus support the longstanding idea that the cortical actin meshwork acts as a physical barrier to exocytosis in secretory cells that prevents unwanted release of toxic granule components at the steady-state [62]. Cellular activation then induces Coro1a-dependent local disassembly of cortical F-actin that creates permissive regions at the cell membrane, allowing for efficient docking and fusion of secretory granules with the plasma membrane. Enhanced levels of cortical F-actin and ineffective actin-depolymerization in *Coro1a*^−/−^ neutrophils or pharmacologic stabilization of F-actin thus hindered neutrophil degranulation, while, conversely, disruption of the cortical F-actin network greatly potentiated granule exocytosis. A role for Coro1a in promoting destruction in F-actin density has also been reported for NK cells, where Coro1a-mediated local clearance of F-actin is required for lytic immune synapse function [35]. Interestingly, the regulatory function of Coro1a on the actin cytoskeleton appears to be highly cell type- and/or context-dependent, as Coro1a deficiency in mast cells is associated with increased degranulation, which has been attributed to an F actin-bundling/stabilizing function of Coro1a in this particular cell type [34].

Noteworthy, while the enhanced cortical F-actin network in *Coro1a*^−/−^ neutrophils was associated with impaired neutrophil degranulation, use of a small molecule inhibitor of Arp2/3 complex-mediated actin nucleation/branching surprisingly also blocked neutrophil granule secretion. Our data, thus, revealed both positive and negative regulatory functions of actin polymerization dynamics on granule exocytosis in neutrophils. These divergent and seemingly contradictory roles of the actin cytoskeleton on neutrophil degranulation are likely attributable to the high complexity of secretory processes that involve multiple discrete steps at different subcellular compartments. While on the one hand Coro1a-mediated local disassembly of cortical F-actin is required to allow granule access to the membrane and fusion pore formation, other steps in the process of granule exocytosis seem to be dependent on active actin assembly. Such processes likely include Arp2/3-mediated granule mobilization and intracellular vesicle transport, as recently shown in the intestinal epithelium [63] and/or Arp2/3-dependet force generation for secretory vesicle expulsion, as reported for Drosophila salivary glands [64].

Neutrophil degranulation, particularly release of primary and tertiary granules, has been shown to depend on Rac2 [65,66], thus, the observed defects in Rac activation in *Coro1a*-deficient neutrophils likely also contribute to impaired degranulation. Moreover, given the prominent role of Rac-GTPases in neutrophil trafficking [67], defective Coro1a/Rac-mediated regulation of actin cytoskeletal dynamics may also underly the previously reported impaired migratory capacity of *Coro1a*-deficient neutrophils [38].

In addition to liberating a potent mix of intragranular mediators, neutrophil degranulation is also interrelated with the generation of ROS. Most of the Cyt_b588_ (p22^phox^ and gp91^phox^) is associated with secondary and tertiary granules in resting neutrophils and only delivered to the membrane upon activation-induced granule exocytosis [68,69]. Also, myeloperoxidase (MPO) from primary granules potentiates the cytotoxic features of ROS by producing highly bactericidal oxidants that can damage nucleic acids, proteins and membranes [70]. Thus, impaired degranulation may also contribute to reduced ROS production in *Coro1a*-deficient neutrophils by limiting the delivery of Cyt_b588_ and MPO. ROS produced by the NADPH oxidase again can activate granular protease and induce the release of NETs. Recent reports on the involvement of actin cytoskeletal rearrangements or Coro1a in NET formation [18,71] may thus at least be partially related to effects on ROS production.

In summary, we have shown that the dynamic restructuring of the actin cytoskeleton mediated by Coro1a serves as a fundamental mechanism to control of neutrophil effector functions, such as oxidative burst formation and cellular degranulation. Our data suggest that a Coro1a/Rac2 signaling hub acts as a central regulatory element that coordinates the recruitment of the components of the NADPH oxidase to the membrane, thereby forming a functional ROS-generating signaling complex. Coro1a-mediated local F-actin remodeling at the plasma membrane, potentially also influenced by the Coro1a/Rac axis, is simultaneously required to provide the docking sites for neutrophil granule alignment, membrane fusion and subsequent release of granule contents.

Given the high similarity between mouse and human Coro1a, our studies suggest that in addition to the reported defects in T cell function, impaired neutrophil effector functions may also contribute to the severe immunodeficiency in patients with Coro1a-mutations, thus expanding the range of clinical manifestations of neutrophil-mediated disease. Moreover, fine-tuning Coro1a activity by targeting either a specific functional arm of Coro1a or one of its interactors to control deleterious neutrophil effector functions may open up novel perspectives for therapeutic approaches.

## 4 Materials and methods

**Mice**. *Coronin 1a*-deficinent (*Coro1a*^−/−^) mice [30] were backcrossed for >10 generations onto C57BL/6N mice. C57BL/6N mice were used as wild-type (WT) controls. All mouse lines originally entered the animal facility of Hannover Medical School via embryo transfer, and were then housed in individually ventilated cages (IVC) under specific pathogen-free (SPF) conditions. For experiments, age-matched 8- to 14-week-old male mice were used. All animal work complied with institutional guidelines and was approved by the local authorities (Lower Saxony Federal State Office for Consumer Protection and Food Safety).

**Antibodies and reagents.** Fluorescent labeled antibodies specific for Ly6G, c-kit, CXCR2, CD63, CD4, CD8, B220, and Ter119 were all obtained from BioLegend. Antibodies to Arpc2, p40^phox^, gp91^phox^ were purchased from Proteintech. Antibodies to p-p40^phox^ (Thr-154), *p*-Ser^PKC^ (phospho-[Ser] PKC substrate were from Cell Signaling Technology, p-p47^phox^ (Ser-328) was from Abcam. *Anti*-p22^phox^ was obtained from Santa Cruz Biotechnology, anti-HSP Ab was from BD Transduction Laboratories. Antibodies to p47^phox^ and β-actin were obtained from Sigma-Aldrich, anti-Rac1 was from Millipore, and anti-Rac2 Ab was purchased from Invitrogen. Antibodies to Coro1a and Coro1b have been described previously [30]. Zymosan and whole glucan particles (WGP) were obtained from Invivogen. Phorbol-12-myristat-13-acetat (PMA), jasplakinolide, diphenyleneiodonium (DPI), luminol, catalase (from bovine liver), and superoxide dismutase were all obtained from Sigma-Aldrich. Lucigenen was obtained from Merck. NSC 23766 was purchased from Tocris Bioscience. Benproperine (phosphate), latrunculin B, and ML-099 were obtained from Cayman Chemical.

**Neutrophil isolation**. Bone marrow cells were flushed from femurs and tibias of both hind limbs with 5 ml of MACS buffer (PBS, 0.5 % BSA, 2 mM EDTA) using a 27-gauge needle (BD Biosciences). After filtering the bone marrow cell suspension through a 40-μm filter (Starlab), neutrophils were isolated by immunomagnetic negative selection procedure using Neutrophil Isolation kit (Miltenyi Biotec, 130-097-658).

**Cultivation of *Candida albicans*.***C. albicans* strain SC5314 (ATCC MYA-2876) was recovered from a −80 °C glycerol stock on a yeast peptone dextrose (YPD) agar plate at 30 °C. Overnight colonies were sub-cultured in YPD medium and grown overnight at 30 °C with shaking at 180 rpm. Fungal cultures were then washed extensively in PBS, and culture dilutions with OD_595_ ≈ 0.5 (measured with an absorbance microplate reader (Agilent BioTek ELx808, Agilent Technologies)), roughly corresponding to 1 × 10^7^ *C. albicans* CFU/ml, were used as a starting dilution for establishing experimental *C. albicans* cultures. *C. albicans* dimorphic growth was controlled by altering the incubation temperature: 30 °C was used for cultivation of the yeast form, while 37 °C facilitated *C. albicans* switching into the hyphal form.

**Chemiluminescence-based analysis of ROS production.** Neutrophils (3 × 10^5^) suspended in assay medium (DMEM, 10 % FCS, 2 mM l-glutamine, 1x Pen/Strep) were seeded in a flat-bottom 96-well plate and left to settle for 20 min at RT. Where indicated, neutrophils were additionally preincubated with actin- or Rac-modifying drugs for 1 h at RT. Neutrophils were then supplemented with a chemiluminescent agent: To visualize total ROS production, luminol (100 μM) was added. Luminol supplemented with superoxide dismutase (SOD, 300 U/ml) and catalase (200 U/ml) was used to measure ROS produced intracellularly. Cell-impermeable lucigenin (100 μM) was added to monitor the extracellular ROS signal. Neutrophils were then activated with either 100 ng/ml PMA (Sigma-Aldrich) or ingestible particulate stimuli: serum-opsonized zymosan (size: ∼3–4 μm, Invivogen), or ∼1.5 × 10^6^ CFU/well of serum-opsonized *C. albicans* yeast (size: ∼4–6 μm). As particulate stimuli that cannot be phagocytosed, 60 μg/well serum-opsonized whole glucan particles (WGP; size: >20 μm, Invivogen), or ∼2 × 10^5^ CFU/well of serum-opsonized *C. albicans* hyphae (length: >20 μm, grown for 2 h in HBSS with 2 % mouse serum at 37 °C) were utilized. Opsonization was achieved by incubating zymosan and WGP for 30 min, and C. albicans yeast for 15 min with 20 % (v/v) of fresh WT mouse serum at 37 °C. As additional stimulus, plate-bound immobilized immune complexes (iIC) were utilized. iIC were formed by coating plates o/n with 3 % BSA in PBS. After washing 3x with PBS, wells were incubated with anti-BSA Ab (mouse mAb; clone BSA-33; Sigma-Aldrich) in PBS for 2 h, followed by 3 additional washing steps. After induction of cell stimulation, the ROS-triggered chemiluminescence signal was detected using an Orion L Microplate Luminometer MPL4 (Titertek-Berthold). The oxidative burst signal was recorded as a number of relative light units (RLU), with a measurement pace of every 125 s over 2 h for all stimuli.

**Flow cytometry-based analysis of ROS production (DHR123 assay).** For the DHR123 assay, 1.5 × 10^5^ MACS-isolated neutrophils suspended in HHBS buffer were seeded into 96-well plates and loaded with 2 μM of DHR123 for 45 min at 37 °C. The cells were then activated with 100 ng/ml PMA for 15 or 30 min at 37 °C. After detaching the cells with Accutase, and washing with PBS, neutrophils were subjected to flow cytometric analysis for ROS production.

**Flow cytometry.** Single cell suspensions of bone marrow cells or neutrophils incubated with TruStain FcX™ (BioLegend) to block Fc Receptors (CD16/32). Cells were subsequently stained with surface-labeled fluorochrome-conjugated antibodies for 30 min. To label the dead cells, eBioscience™ Fixable Viability Dye eFluor™ 780 (Thermo Fisher Scientific) diluted in PBS was applied to samples for 10 min. For intracellular staining, the cells were fixed in 4 % PFA for 20 min at RT and permeabilized for 5 min with 0.2 % Triton X-100 in PBS. The relative content of F-actin was measured by staining with Alexa Fluor 647-conjugated phalloidin (Thermo Fisher Scientific) for 30 min. Samples were subjected to flow cytometric analysis employing a BD FACSCanto II analyzer (BD Biosciences, San Jose, CA, USA) operated by BD FACSDiva software (ver. 6.1.3; BD Biosciences). Data were analyzed with FlowJo software (ver. 10; BD Biosciences).

**Phagocytosis assay.** 2.5 × 10^5^ neutrophils were incubated for the indicated times at 37 °C in a 96-well plate with FITC-labeled serum opsonized zymosan (FITC-SOZ) at an effector:target ratio of 1:5 (neutrophils: zymosan particles). As controls, phagocytosis was inhibited by incubation on ice or by treatment with cytochalasin D. The reaction was stopped by placing the samples on ice. The fluorescence of extracellular FITC-SOZ was quenched with Trypan blue and phagocytosis was assessed by flow cytometric measurement of the percentage of FITC-positive live neutrophils.

**ELISA-based degranulation assay.** Exocytosis of neutrophil granule subsets was measured using ELISA kits from R&D Systems. Specifically, release of primary granule contents was determined by ELISA for myeloperoxidase (MPO; mouse total myeloperoxidase DuoSet® ELISA, DY3667). Exocytosis of secondary granules was assessed by ELISA for neutrophil gelatinase-associated lipocalin (NGAL; mouse total lipocalin-2/NGAL DuoSet® ELISA, DY1857). ELISA for matrix metalloproteinase-9 (MMP-9; mouse total MMP-9 DuoSet® ELISA, DY6718) was used to reflect release of tertiary granules. ELISA kits were used according to the manufacturer's procedure utilizing 96-well microplates (Sarstedt, 82.1581.200). To identify the total amount of the tested granule proteins, 0.3 × 10^6^ neutrophils were treated with 1 % Triton X-100 in PBS for 60 min at 37 °C. Optical density was recorded with a microplate reader (Agilent BioTek ELx808*, Agilent Technologies)* at 450 nm and 595 nm. Gen5™ Microplate Reader Software (ver. 2.07; BioTek) was used for 450–595 nm wavelength correction and data processing.

**Immunofluorescence microscopy.** Neutrophils (2.5 × 10^5^) were seeded into channels of poly-l-lysine-coated VI^0.4^ μ-slides (ibidi) and allowed to settle for 30 min at 37 °C. The cells were activated with TNFα (20 ng/ml) for 30 min at 37 °C, fixed with 4 % paraformaldehyde for 20 min at RT, permeabilized with 0.2 % Triton X-100 for 5 min at RT, and further treated with 0.1 % saponin in PBS buffer (eBioscience). After blocking unspecific staining in a buffer containing 0.5 % BSA for phalloidin staining, or Armenian hamster IgG for Coro1a detection, cells were stained with Alexa Fluor 488- or Alexa Fluor 546-conjugated phalloidin (Invitrogen), and DyLight 649-conjugated mAb anti-Coro1a (clone 5E10.3.7, Armenian hamster IgG [30], in 0.1 % saponin/PBS buffer. MMP-9 was detected with biotinylated anti-MMP9 polyclonal Ab (R&D Systems, BAF909) followed by staining with Streptavidin-AF488 (Thermo Fisher Scientific, S11223). Nuclear counterstain was achieved with 300 nM DAPI (Thermo Fisher Scientific) for 5 min at RT. Images were acquired through a Zeiss LSM 980 laser scanning confocal microscope with Airyscan 2 (ZEISS Microscopy) and analyzed by FIJI software (ver. 1.53u).

**Transmission electron microscopy (TEM).** For TEM, samples were prepared as described previously [72]. TNFα- or mock-stimulated mature neutrophils from WT or *Coro1a*^−/−^ mice were incubated in EM fixative solution (1.5 % PFA, 1.5 % glutaraldehyde, 0.15 M HEPES, pH 7.35) for 30 min at RT. The cells were then immobilized in 2 % agarose, and postfixed in 1 % osmium tetroxide. After embedding in Epon resin, sections of 60-nm were transferred onto formvar-coated copper grids. Neutrophil sections were viewed employing TEM Morgagni 268 (Philips/FEI Electron Optics) and iTEM software (Olympus SIS) used for image acquisition.

**Rac activation assay.** Rac activation was assessed by using the Rac1 Activation Magnetic Beads Pulldown Assay kit (Merck) according to the manufacturer's instructions. Briefly, 2 × 10^6^ WT or *Coro1a*^−/−^ neutrophils were suspended in HHBS buffer and activated with PMA (100 ng/ml) for the indicated times, or left untreated for 2 min. The reaction was terminated by addition of 3.6x Mg^2+^ lysis/wash buffer (MLB) and transfer of the samples on ice. Insoluble debris was cleared by centrifugation (5 min, 14000×*g*). Aliquots of the extract (10 %) were used as a total Rac loading control. Active Rac1 was pulled down from the remaining lysate with the Rac-binding domain of PAK1 conjugated to magnetic beads by use of a magnetic concentrator DynaMag™-2 (Thermo Fisher Scientific). Beads were washed 3x with MLB buffer, followed by protein elution with Laemmli buffer. Activated and total Rac proteins were examined by SDS-PAGE and Western blotting.

**Rac translocation assay.** In order to evaluate Rac translocation, subcellular fractionation of neutrophils was carried out using the “Cell Fractionation Kit” (Cell Signaling Technology), according to the manufacturer's manual, with minor adjustments. Briefly, 2.5 × 10^6^ of WT or *Coro1a*^−/−^ neutrophils were suspended in HHBS buffer and stimulated with PMA (100 ng/ml, 10 min, 37 °C) or left unstimulated. After stimulation, neutrophils were detached with AccutaseTM treatment (5 min at RT), washed with ice-cold PBS and fractionated into the Whole Cell Lysate (WCL), the Cytoplasmic Fraction (CF), and the Membrane and Organelle Fraction (MOF). Immediately before the fractionation, Cytoplasm Isolation Buffer and Membrane Isolation Buffer were supplemented with 1x Protease Inhibitor Cocktail from the kit, and 1 mM PMSF. For the WCL samples, the cell suspension was aliquoted and used as an unfractionated control. To acquire the CF, the cell suspension was centrifuged at 500×*g* for 5 min at 4 °C, and the pellet was resuspended in Cytoplasm Isolation Buffer buffer, vortexed for 5 s, and incubated for 5 min on ice. The resulting protein extract was centrifuged for 5 min at 500×*g* at 4 °C, and the supernatant, representing the Cytoplasmic Fraction, was collected. To isolate the MOF, the pellet was resuspended in the Membrane Isolation Buffer, vortexed for 15 s, and incubated for 5 min on ice. After centrifuging the resulting extract for 5 min at 8000×*g*, supernatant contained the Membrane and Organelle Fraction. Samples were analyzed by SDS-PAGE and Western blotting.

**Co-immunoprecipitation and analysis of mass-spectrometry (Co-IP/MS).** Purified neutrophils from WT and *Coro1a*^−/−^ mice were either activated with 100 ng/ml PMA for the indicated times or left untreated at 37 °C. The reaction was terminated by centrifugation at 4 °C. The cell pellet was lysed with IGEPAL® CA-630-based lysis buffer (10 Tris-HCl, pH 7.4, 10 mM MgCl2, 10 mM KCl, 150 mM NaCl, 0.5 % (v/v) IGEPAL® CA-630, 1 mM EDTA) supplemented with protease and phosphatase inhibitors (complete Protease Inhibitor Cocktail containing with1 mM PMSF, 10 mM NaF, 1 mM Na3VO4, 1 μM Pepstatin A) for 20 min on ice. Co-IP was conducted by incubation of cell lysates with magnetic beads conjugated to either rabbit polyclonal anti-Coro1a antibody (Proteintech) or isotype matched control antibody in an orbital rotator Intelli-Mixer RM-2M for 2 h at 4 °C. Bead-associated protein complexes were collected by a use of a DynaMagTM-2 magnetic particle concentrator (Invitrogen Dynal AS). After washing, beads suspension was magnetically separated from the flow-through. For elution of the protein complex-bound beads, reducing Laemmli buffer was added and heated for 5 min at 95 °C. Samples were subjected to SDS-PAGE and Western blotting using specific antibodies. For the analysis via MS, samples were supplemented with acrylamide (final concentration: 2 % (w/v)) to alkylate cysteine residues. Following SDS-PAGE and Coomassie staining, each lane of the Coomassie dye-stained gel was excised, sliced into smaller gel pieces and further processed as previously described [73]. Briefly, gels were destained, dehydrated, and in-gel digested with trypsin (o/n, 37 °C). Following termination of digestion, the peptide extracts were vacuum concentrated. Peptide extracts were resolubilized in mobile phase for HPLC (2 % acetonitrile (w/v) and 0.1 % trifluoroacetic acid (v/v) in water), centrifuged at 20000×*g* and aliquoted. Peptides were then separated and analyzed via shotgun approach employing the nanoflow ultra-high-pressure liquid chromatography system (RLSC, Thermo Fisher Scientific) hyphenated to the mass spectrometer (Orbitrap Exploris 240, Thermo Fisher Scientific). Acquired MS data were analyzed utilizing MaxQuant software (ver. 1.5; (Cox and Mann, 2008)), Perseus software (ver. 2.0.6.0 (Tyanova et al., 2016);), and mouse entries of Uniprot database (FDR <0.01 on protein and peptide level).

**Western blotting.** Whole cellular lysates were prepared in cell extraction buffer (1 % (v/v) IGEPAL® CA-630, 50 mM Tris-HCl (pH = 7.4), 150 mM NaCl, 10 mM NaF, 1 mM Na_3_VO_4_, 1x cOmplete Protease Inhibitor Cocktail, 1 mM PMSF, 1 mM EDTA, 1 mM EGTA, 1 μM Pepstatin A) for 20 min on ice. Whole cellular lysates, immunoprecipitates or subcellular fractions prepared with the “Cell Fractionation Kit” (Cell Signaling Technology) (see above) were resolved by SDS-PAGE and proteins were detected by Western blotting using the indicated antibodies and HRP-conjugated secondary reagents (Jackson ImmunoResearch). Signals were detected using WesternBright™ Sirius HRP substrate (Advansta) and recorded using an INTAS ChemoStar ECL Imager (*INTAS Science Imaging Instruments GmbH*).

**Real-time quantitative RT-PCR analysis.** Total RNA was purified from neutrophils using the RNeasy Micro kit (QIAGEN) and reverse transcribed using the First Strand cDNA Synthesis Kit (Thermo Fischer Scientific). For real-time quantitative RT-PCR, gene expression of coronin family members was measured relative to Hprt using the Universal Probe Library (Roche) TaqMan-based system (Applied Biosystems). Primers and Universal ProbeLibrary ID numbers have been described previously [34]. Amplification was performed in a fluorescence temperature cycler (LightCycler 480; Roche).

**Statistical analysis**. All statistical data analysis was performed with and visualized using GraphPad Prism (ver. 9.0.0; Dotmatics). To analyze statistical significance (P < 0.05), unpaired Student's t-test or two-way ANOVA was employed. Experiments were repeated at least three times independently.

## CRediT authorship contribution statement

**Anton Shaverskyi:** Visualization, Methodology, Investigation, Formal analysis, Conceptualization. **Jan Hegermann:** Methodology, Investigation. **Korbinian Brand:** Resources. **Kyeong-Hee Lee:** Writing – review & editing, Writing – original draft, Supervision, Funding acquisition, Conceptualization. **Niko Föger:** Writing – review & editing, Writing – original draft, Visualization, Supervision, Investigation, Funding acquisition, Conceptualization.

## Declaration of competing interest

The authors declare no competing interests.

## Data Availability

Data will be made available on request.
