# Supplementary material for: Coronin 1a-mediated F-actin disassembly controls effector function in murine neutrophils
Source: Redox Biol. 2025 Mar 26;82:103618. doi: 10.1016/j.redox.2025.103618 (PMC11997354; doi:10.1016/j.redox.2025.103618)

Supplemental Figure Legends

Supplemental Figure S1

Coro1a deficiency does not affect neutrophil development

(A) Expression of the seven mammalian coronin family members in purified mouse neutrophils was detected via quantitative real-time PCR relative to HPRT1. Data are mean ± SD; n = 2-3. **(B)** Western blot analysis showing expression of immunologically relevant coronin proteins in WT and *Coro1a*$^{-/-}$ (KO) neutrophils. Data are representative for 5 independent experiments. (**C**) Representative flow cytometric gating strategy to identify developing and mature BM neutrophil subpopulations. R1: lineage-negative (CD4^{neg}CD8^{neg}B220^{neg}Ter119^{neg}) population; R2: eosinophiles; R3: neutrophil subpopulations; G1: neutrophil progenitors; G2: pre-neutrophils, G3: immature neutrophils, G4: mature neutrophils. (**D**) Percentage and **(E)** absolute neutrophil count (right) of developing and mature WT and *Coro1a*$^{-/-}$ (KO) bone marrow neutrophil subpopulations G1-G4 from 8-week-old male mice**.** Data are mean ± SD (n = 9 mice) of two independent experiments. Unpaired Student's t-test: ns > 0.05, * < 0.05.

Supplemental Figure S2

Impaired ROS production but normal expression of NADPH oxidase subunits in *Coro1a*-deficient neutrophils

(A) Dose-response experiment showing total integrated ROS measured with luminol chemiluminescence in WT and *Coro1a*$^{-/-}$ (KO) neutrophils stimulated with titrated concentrations of PMA. **(B, C)** Flow cytometry-based measurement of PMA (100 ng/ml)-induced intracellular ROS generation in neutrophils using dihydrorhodamine 123 (DHR123). **(B)** Representative histogram of rhodamine 123 (Rh123) profile at 15 min of stimulation. US, unstimulated. n = 2 **(C)** Fluorescence intensity of Rh123 at the indicated time points in WT and *Coro1a*$^{-/-}$ (KO) neutrophils. Data show GeoMFI ± SD. DPI (diphenyleneiodonium chloride) specifically inhibits NADPH oxidase-derived ROS. **(D)** Western blot-based expression analysis of NADPH oxidase subunits and Rac proteins in lystes of WT and *Coro1a*$^{-/-}$ (KO) isolated from two separate mice of each genotype. (**E**) Western-blot showing PMA-induced phosphorylation of p47^{phox} on Ser-328 in WT and *Coro1a*$^{-/-}$ (KO) neutrophils. **(A, C)** Unpaired Student's t-test: * < 0.05, **** < 0.0001. Data are representative for 2-3 independent experiments.

Supplemental Figure S3

Reduced phagocytosis in *Coro1a*-deficient neutrophils

Phagocytosis of FITC-labeled serum-opsonized zymosan (FITC-SOZ) by WT and *Coro1a*$^{-/-}$ (KO) neutrophils at an effector:target (E:T) ratio of 1N:5Z was measured by flow cytometry (N: neutrophil; Z: zymosan). Extracellular FITC-SOZ signal was quenched with Trypan blue. As control, internalization was inhibited by keeping neutrophils on ice or by treatment with cytochalasin D. Data are mean ± SD (n = 2). Data are representative for three independent experiments. Unpaired Student's t-test: ns > 0.05, * < 0.05, ** < 0.01.

Supplemental Figure S4

Impaired granule exocytosis in *Coro1a*-deficient neutrophils

Release of **(A)** neutrophil gelatinase-associated lipocalin (NGAL) from secondary granules or **(B)** matrix metalloproteinase-9 (MMP-9) from tertiary granules into culture supernatants upon stimulation of neutrophils with PMA (100 ng/ml). Data are mean ± SEM. 2-way ANOVA test (Sidak). Data are representative for at least 3 independent experiments. **(C)** Transmission electron microscopy (TEM) analysis of WT and *Coro1a*$^{-/-}$ (KO) neutrophils either unstimulated or challenged with TNFα (30 min). Data show quantification of neutrophil secondary – quaternary (2°G – 4°G) granule numbers per cellular section. Data are mean ± SEM (n = 7-9). one-way ANOVA, Tukey's multiple comparisons test: ns > 0.05, * < 0.05, ** < 0.01. **(D)** Airyscan-enhanced confocal micrographs of WT and *Coro1a*$^{-/-}$ (KO) neutrophils that were stimulated with TNFα for 30 min. Neutrophils were fixed permeabilized and stained for F-actin (phalloidin) and MMP-9. Scale bar: 3 µm.

Supplemental Figure S5

Viability of WT and *Coro1a*-deficient neutrophils is not affected by actin-cytoskeleton modulating or Rac-targeting drugs

(A) Viability of WT and *Coro1a*$^{-/-}$ (KO) neutrophils after 60 min incubation with either latrunculin B (Lat, 1 µM; actin-depolymerizing agent), jasplakinolide (Jas, 16 µM; actin-stabilizing agent), or benproperine phosphate (Benp, 30 µM; Arp2/3-complex inhibitor). **(B)** Viability of WT and *Coro1a*$^{-/-}$ (KO) neutrophils after 60 min incubation with 150 µM NSC23766 (NSC; Rac inhibitor) or 10 µM ML-099 (ML; pan Ras activator). Effects of ML-099 were controlled by neutrophils incubated in carrier (DMSO)-containing medium. Data are mean ± SD (n = 2). Unpaired Student's t-test, ns > 0.05. Data are representative for at least 2 independent experiments.

Supplemental Figure 1

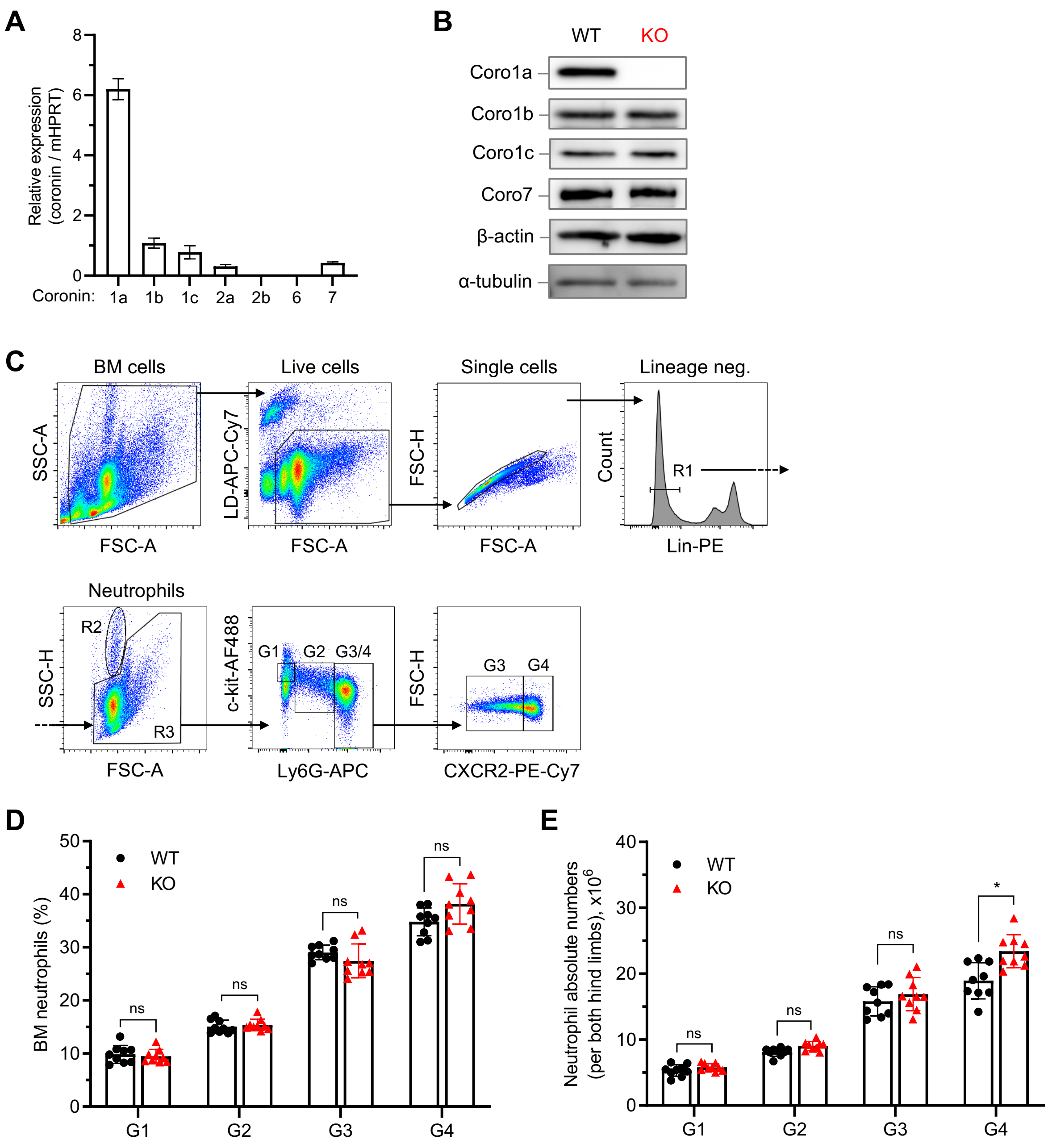

Supplemental Figure 2

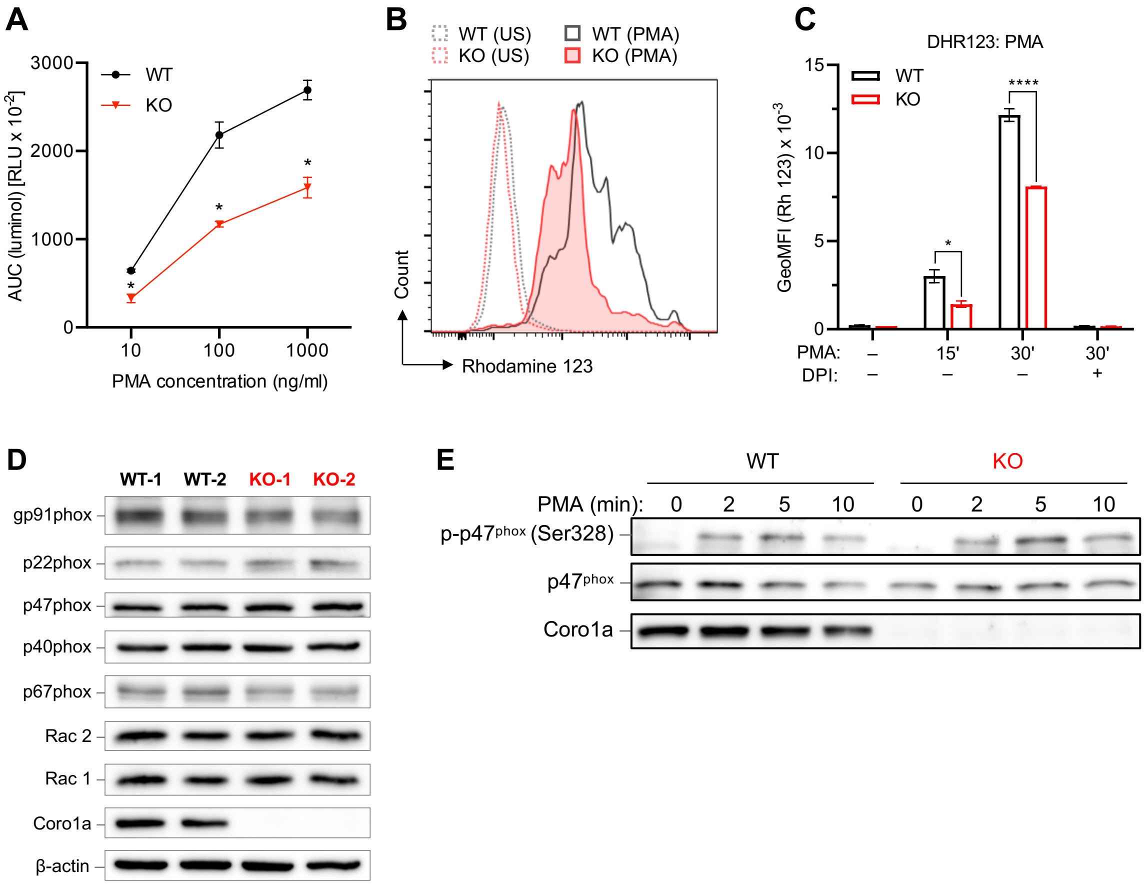

Supplemental Figure 3

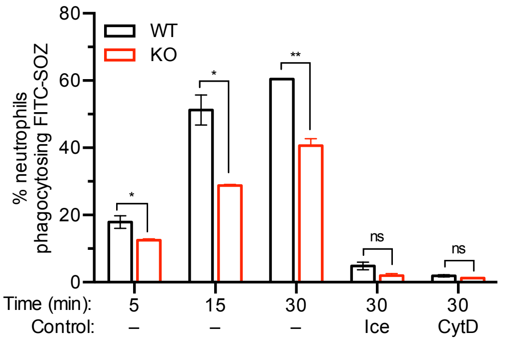

Supplemental Figure 4

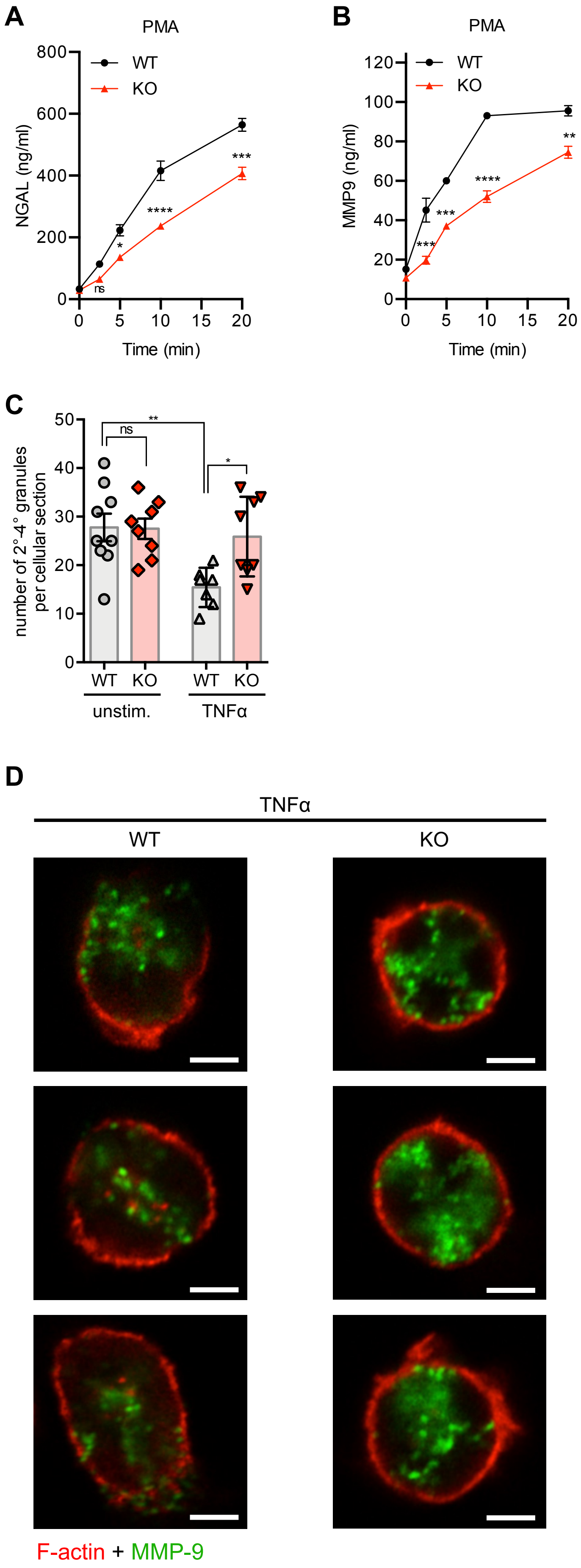

Supplemental Figure 5

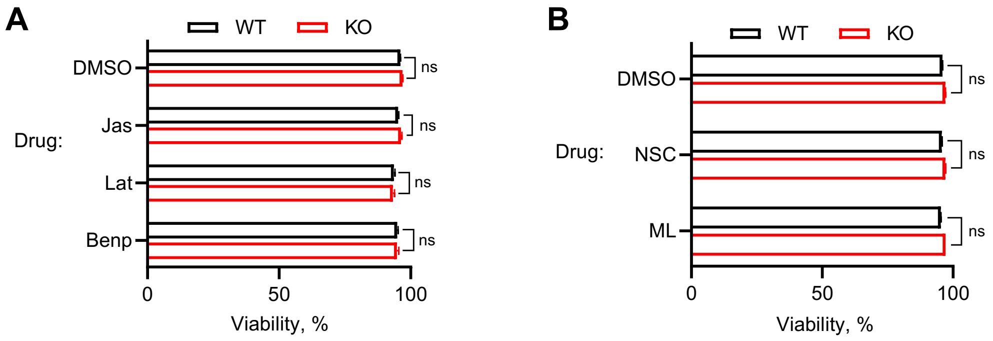

Supplement: Multimedia component 1 [file mmc1.pdf]
